# Supplementary material for: Identification of photoperiod-induced specific miRNAs in the adrenal glands of Sunite sheep (Ovis aries)
Source: Front Vet Sci. 2022 Jul 22;9:888207. doi: 10.3389/fvets.2022.888207 (PMC9354845; doi:10.3389/fvets.2022.888207)
Supplement: Supplementary file 1 [file Data_Sheet_1.ZIP › Supplementary file/miRDeep2 file.zip!/miRDeep2 file/Know_Structure/LP42A1/oar-mir-485.pdf]

miRBase precursor : oar-mir-485
Total read count : 1278
oar-miR-485-5p read count : 461
oar-miR-485-3p read count : 816
remaining reads : 1

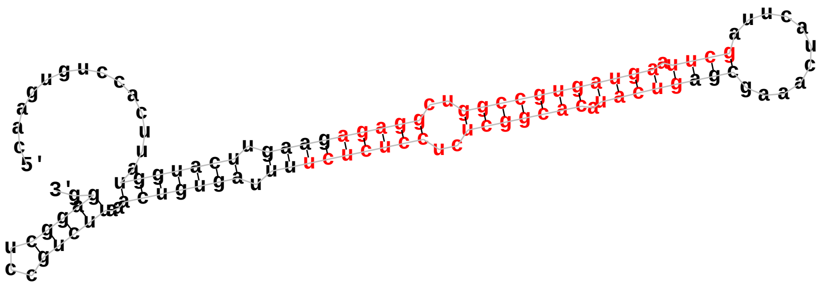

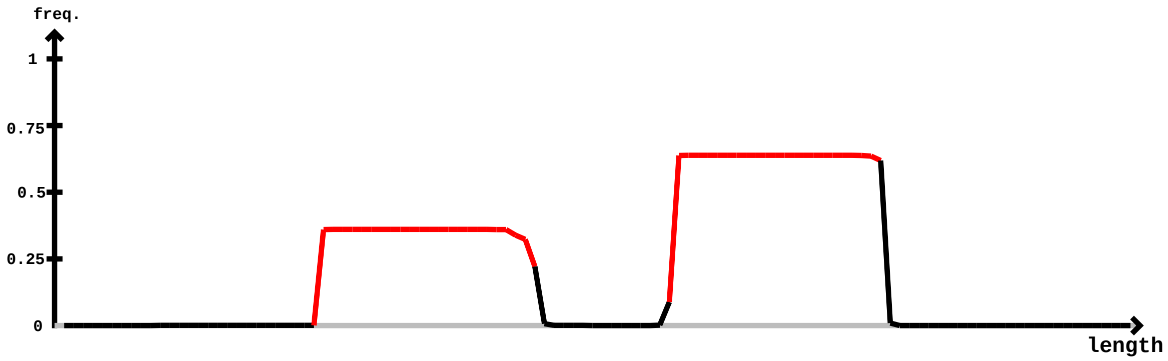

oar-miR-485-5p

oar-miR-485-3p

```
5'- caaguguccacuuaugguacuugaagagaggcuggccgugaugaauucgauucaucaaagcgagucauacacggcucuccucucuuuuagugucaaauucugccucggagg   -3'    exp
    ((((((.(((....)))))))))((((((((..((((((((((.((((...........)))))))).)))))))..))))))))..........(((((...))))).    reads    mm    sample
    .........acuuaugguacuugaag.....................................................................................    1    0    seq
    ..........................agaggcuggccgugauga...................................................................    1    0    seq
    ..........................agaggcuggccgugaugaau.................................................................    27    0    seq
    ..........................agaggcuggccgugaugaauu................................................................    20    0    seq
    ..........................agaggcuggccgugaugaauuc...............................................................    130    0    seq
    ..........................agaggcuggccgugaugaauucg..............................................................    273    0    seq
    ..........................agaggcuggccgugaugaauucga.............................................................    8    0    seq
    ..........................agaggcuggccgugaugaauucgauuca.........................................................    1    0    seq
    ...........................gaggcuggccgugaugaauucg..............................................................    1    0    seq
    .............................................................gagucauacacggcucuccucuc...........................    1    0    seq
    .............................................................gagucauacacggcucuccucucu..........................    1    0    seq
    ..............................................................agucauacacggcucuccucuc...........................    3    0    seq
    ..............................................................agucauacacggcucuccucucu..........................    107    0    seq
    ..............................................................agucauacacggcucuccucucuu.........................    1    0    seq
    ...............................................................gucauacacggcucuccuc.............................    1    0    seq
    ...............................................................gucauacacggcucuccucu............................    3    0    seq
    ...............................................................gucauacacggcucuccucuc...........................    17    0    seq
    ...............................................................gucauacacggcucuccucucu..........................    670    0    seq
    ...............................................................gucauacacggcucuccucucuu.........................    11    0    seq
    ................................................................ucauacacggcucuccucucu..........................    1    0    seq
```
